# Changes in roll-your-own tobacco and cigarette sales volume and prices before, during and after plain packaging legislation in the UK

**DOI:** 10.1136/tobaccocontrol-2018-054734

**Published:** 2019-05-09

**Authors:** Magdalena Opazo Breton, John Britton, Ilze Bogdanovica

**Affiliations:** 1 UK Centre for Tobacco and Alcohol Studies, Epidemiology and Public Health, University of Nottingham, Nottingham, UK; 2 Division of Epidemiology and Public Health, University of Nottingham, Nottingham, UK; 3 UK Centre for Tobacco Control Studies, Nottingham, UK

**Keywords:** plain packaging, prices, roll-your-own tobacco

## Abstract

**Background:**

Plain packaging and minimum pack size legislation for tobacco products was introduced in the UK in May 2016, with a 1-year sell-off period until May 2017, during which both fully branded and plain packs of various sizes were legally available. This study investigates trends in prices of roll-your-own tobacco (RYO) before, during and after implementation of this legislation, and compares trends with those observed in the cigarette market.

**Methods:**

We used Nielsen Scantrack data for the period from March 2013 to June 2018 to describe trends in UK inflation-adjusted prices and volumes of both RYO and cigarettes, and linear regression to estimate changes in prices associated with the introduction of plain packaging and the minimum pack sizes of 30 g RYO and 20 cigarettes.

**Results:**

In contrast to a downward trend in cigarette sales volumes, RYO volumes rose throughout the study period. By the time plain packs accounted for 75% or more of sales, the average price of products sold in equivalent pack sizes had increased, relative to average prices in the year before implementation and with adjustment for tax changes, from 34.9 to 38.8 pence per gram for RYO (mean difference 4.26, 95% CI 3.99 to 4.53 pence, 12% increase), and from 38.6 to 41.13 pence for cigarettes (mean difference 2.53, 95% CI 2.24 to 2.83 pence, 7% increase) per cigarette.

**Conclusions:**

New legislation resulted in higher prices for RYO and manufactured cigarettes. However, sales volumes of RYO continued to increase throughout the study period, perhaps because RYO remains a less expensive means of smoking tobacco.

## Introduction

In December 2012 Australia became the first country in the world to require all tobacco products to be sold in plain packs,^1^ and in 2016 was followed by the UK and France.^2 3^ The UK legislation was implemented in conjunction with measures required by the 2014 European Union Tobacco Products Directive^4^ and incorporated a 12-month sell-off period. Thus, the legislation required all tobacco products manufactured or imported to the UK market after 20 May 2016, and from 20 May 2017 all products sold in the UK, to be in plain packs of at least 20 cigarettes or 30 g roll-your-own tobacco (RYO).^2^ Several other countries, including New Zealand, Ireland and Norway, have since implemented, or are implementing, similar legislation.^3^

In Australia, the introduction of plain packaging was associated with changes in perception of packaging,^5^ increased quitting^6^ and an increase in prices paid for tobacco products.^7^ However there has been little evaluation of the effect of plain packaging on prices and consumption outside of Australia. We have previously reported the effect of the UK legislation on cigarette product diversity and prices in the UK, demonstrating that in the lead-up to the implementation period the tobacco industry introduced many new and low-price products in packs of less than 20 cigarettes, and that the transition to plain packs of at least 20 cigarettes was associated with a significant increase in cigarette prices.^8^ However we did not investigate trends in prices of RYO, to which many price-conscious smokers in the UK turn when manufactured cigarettes become less affordable.^9^ This study uses similar methods to our earlier study^8^ to describe RYO price and sales trends in relation to pack sizes before, during and after the UK transition to plain packaging, and in relation to cigarette sales volume and prices.

## Methods

### Data

We used Nielsen Scantrack data on retail sales of cigarettes and RYO from March 2013 to June 2018. These data included monthly volume of sales, value of sales, units sold and estimated average retail price for products scanned from a sample of 75 000 megastores, superstores, high street stores and convenience stores in the UK as described elsewhere.^8 10^ We included make-your-own cigarette packs in the RYO category.

### Measures

#### Tobacco product definitions

Cigarette products were defined as in our previous study,^8^ and based on that definition RYO products were identified according to the following characteristics:

- Brand: for example, Amber Leaf, Cutter’s Choice or Golden Virginia.
- Brand variant: additional product descriptors, such as ‘Quality Blend’, ‘Additive free’ or ‘Duo Fresh’.
- Pack size: the quantity of tobacco, in grams, contained in each products pouch, such as 4 g, 10 g, 12.5 g, 25 g, 30 g or more.
- Multipack size: for the small proportion of products including more than one pouch of tobacco, the number of pouches in the pack.

Hence a product might be ‘Roll your own Amber Leaf 12.5 grams’, ‘Make your own Ashford Blue 50 grams’ or ‘Roll your own Cutters Choice Original 30 grams, 5 pouches’.

#### Volume of sales

Volume of sales of RYO products was measured in grams, as the sum of grams per pouch multiplied by the number of pouches per pack, for each month. For cigarette products, volume of sales is estimated as the number of sticks (number of cigarettes in a pack multiplied by the number of packs in the multipack) each month.

#### Sales in plain packs

The Nielsen data set specifically identified cigarettes sold in plain packs but did not do so for RYO. However, since few RYOs were sold in 30 g packs before the plain packaging sell-off period began, we inferred that 30 g products introduced during the sell-off period were in plain packs in line with the legal requirements. To study the association between the gradual implementation of the plain packaging legislation and prices, we created a variable called ‘plain pack sales’ which was inferred for RYO and computed for cigarettes using volume of sales. For cigarettes, the variable used the percentage of volume of sales in plain packs sold compared with total volume of sales, while for RYO we used the percentage of volume of sales of packs containing 30 g or more of tobacco compared with total volume of sales. Our variable ‘plain pack sales’ had the value ‘1’ before May 2016 when the implementation period began; ‘2’ from May 2016 until the volume of plain pack sales reached 50% of the total; ‘3’ for the period in which this proportion was between 50% and 75% of the total; and ‘4’ when it exceeded 75% of the total volume of sales.

#### Price

We defined price for RYO products as the average retail price (in pence) per gram, and for cigarette products, pence per cigarette. The Nielsen data set categorised prices as standard or promotional, the latter applying when a price reduction of 5% or more was observed compared with the second highest price registered for the same product during the previous 6 weeks. Our analysis only used standard prices since the number of products with promotional price decreased considerably during the study period (from around 54% to 23% for cigarettes and from 51% to 14% for RYO). Price per gram and price per cigarette were estimated as the arithmetic mean price divided by the number of grams of tobacco or the number of cigarettes per pack.

Since Nielsen created an additional price category for plain pack cigarette products, but not for RYO, we averaged plain pack prices and standard prices for cigarettes to compare effects in RYO and cigarettes using the same outcome variable. All prices were adjusted for inflation using consumer price index specific for tobacco products to create a deflator with a base on March 2013.^11^

#### Tax period

To control for changes in tax levels over time, we constructed a ‘tax period’ variable based on UK tobacco duty rates during the study period,^12^ which typically increased in March each year. For cigarettes it increased yearly until March 2017, but in May 2017 a minimum excise tax was introduced and it was increased in November that year. Our ‘tax period’ variable allowed us to adjust simply for annual changes in tobacco duties, and for an additional one-off increase in minimum duty in November 2017, by grouping together months in which the same duty structures applied and creating step changes every time duty rates were changed. Since these structures differ between cigarettes and RYO tobacco, a separated ‘tax period’ variable was created for each.

### Statistical analysis

We used line graphs to display time trends in total volume of sales by pack size and average retail prices by pack size and price category (standard and promotional). For sales volume we added a linear fit for total volume in order to compare trends between cigarettes and RYO. We graphically displayed the gradual implementation of the policy by plotting time trends for the proportion of volume of sales represented by legal pack size products determined by the policy (30 g or more for RYO and 20 cigarettes or more for cigarettes) and compared it with the proportion of sales represented by plain pack cigarette products to justify our inferred plain pack sales variable for RYO. We then estimated the association between the proportion of plain pack sales volumes and price per gram and per cigarette using linear regression since both price per gram and price per cigarette exhibited an approximately normal distribution in a histogram. We estimated the association using data from 1 year before implementation, the transition year and a year after full implementation (May 2015–May 2018). Full time period from March 2013 was not included in the regression analysis to reduce noise in the model. We used two regression models for both cigarettes and for RYO: the first included all products available on the market (thus accounting for the fact that during the implementation period, the full range of different pack sizes coexisted on the market); and the second, restricted to pack sizes that were legal (30 g or more of RYO and 20 or more for cigarettes) under the plain packaging legislation. The second model included an additional adjustment by brand name to account for those contextual factors that might have affected brands differently since price segmentation by brand has been identified in the literature.^13^ Since the second model only uses products marketed in 30 g packs, there was no need to adjust by product size. Both models were adjusted by our tax period variable to account for time trends and tax changes. All analyses were performed using only single pack products (no multipack products) for two reasons: first, multipack size products have considerably lower price per cigarette and per gram; and second there are relatively few RYO multipack products. We used Stata V.15.1 and statistical significance level was set to 95%.

## Results

### Sales volumes

**Figure 1:**
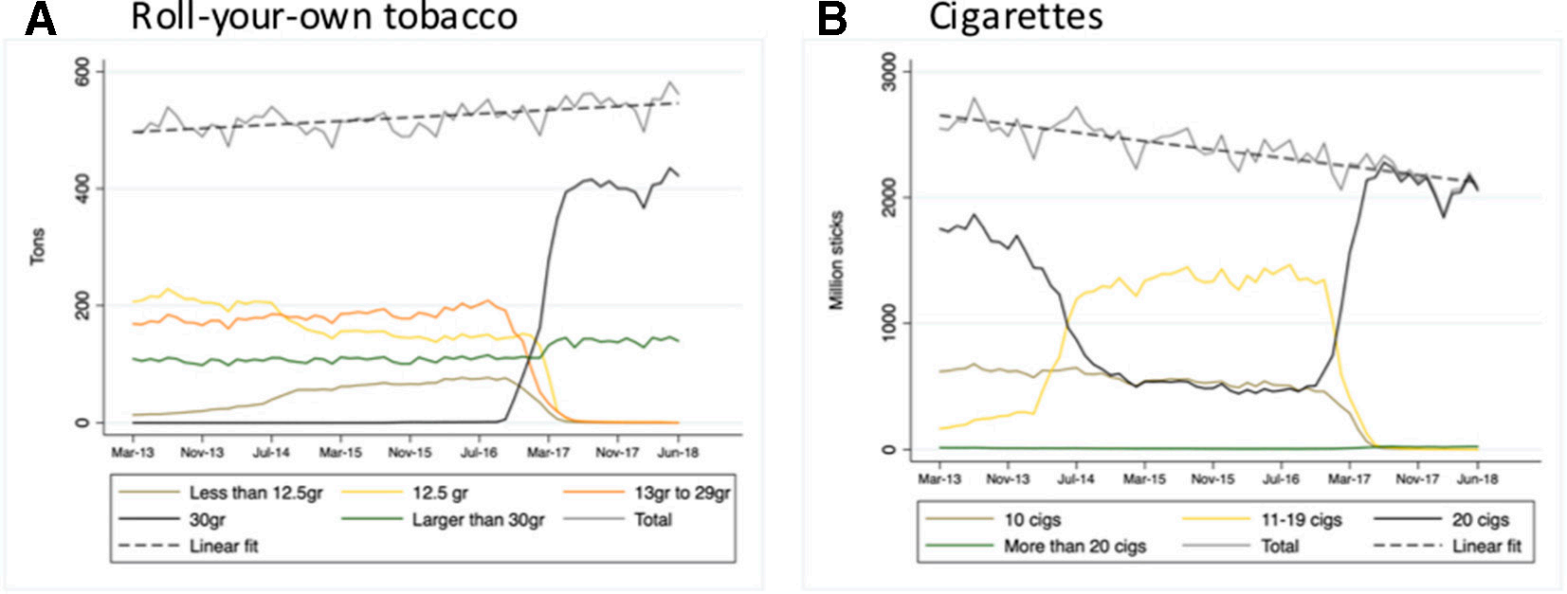
Sales volumes for (A) roll-your-own tobacco and (B) cigarette pack sizes, March 2013–June 2018.

Figure 1 shows that the monthly total volume of RYO sold increased slowly, from 497.1 to 561.8 tons between March 2013 and June 2018 (with an estimated monthly linear increase of 0.16%). Before May 2016 the RYO market was dominated by packs of less than 30 g, and from the beginning of the study period until the early months of the sell-off period there was an increase in volume of sales in the smallest pack size category (less than 12.5 g). Sales volumes in packs larger than 30 g remained relatively constant at around 20% of the total volume of sales before the emergence of 30 g standard packs, and rose slightly with the transition to plain packs, mostly from early 2017 (figure 1A). At the start of the sell-off period in May 2016, the volume of RYO sold in 30 g packs was near zero but increased rapidly from August 2016 to around 400 tons per month in May 2017, representing 75% of the total volume of sales by the end of the study period. The relative popularity of smaller RYO pack sizes in the lead-up to plain packaging reflects that of smaller packs of cigarettes, which dominated the cigarette market until early 2017 (figure 1B). In contrast to the upward trend in total sales volumes of RYO, cigarette volumes declined steadily over the study period (with an estimated monthly linear decrease of 0.32%).

### Price diversity

**Figure 2:**
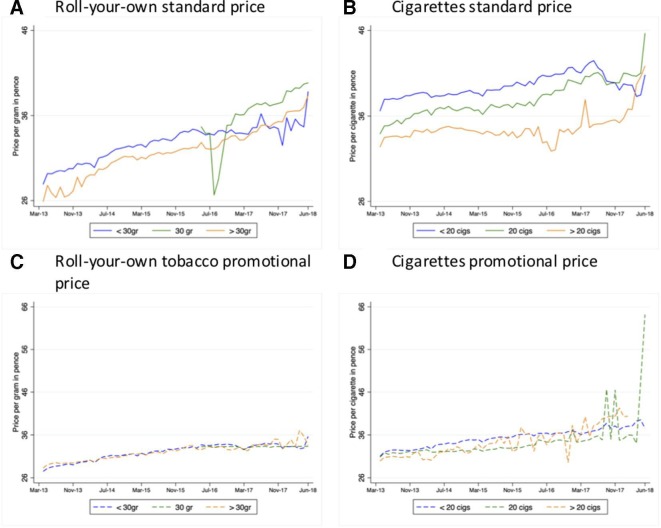
Inflation-adjusted price trends price per gram of roll-your-own tobacco (A, C) and per cigarette (B, D), by pack size, March 2013–June 2018.

In contrast to the previously reported^8^ marked price diversity between cigarettes sold in different pack sizes before the implementation of plain packaging (figure 2B), RYO prices per gram were relatively similar between the various pack sizes throughout the study period, regardless of promotional pricing. The only exception was the new 30 g packs, which were considerably cheaper than other pack sizes when their volume of sales was close to zero, but rose in price as the sell-off period progressed and became the most expensive pack size after April 2017 (figure 2A).

**Figure 3:**
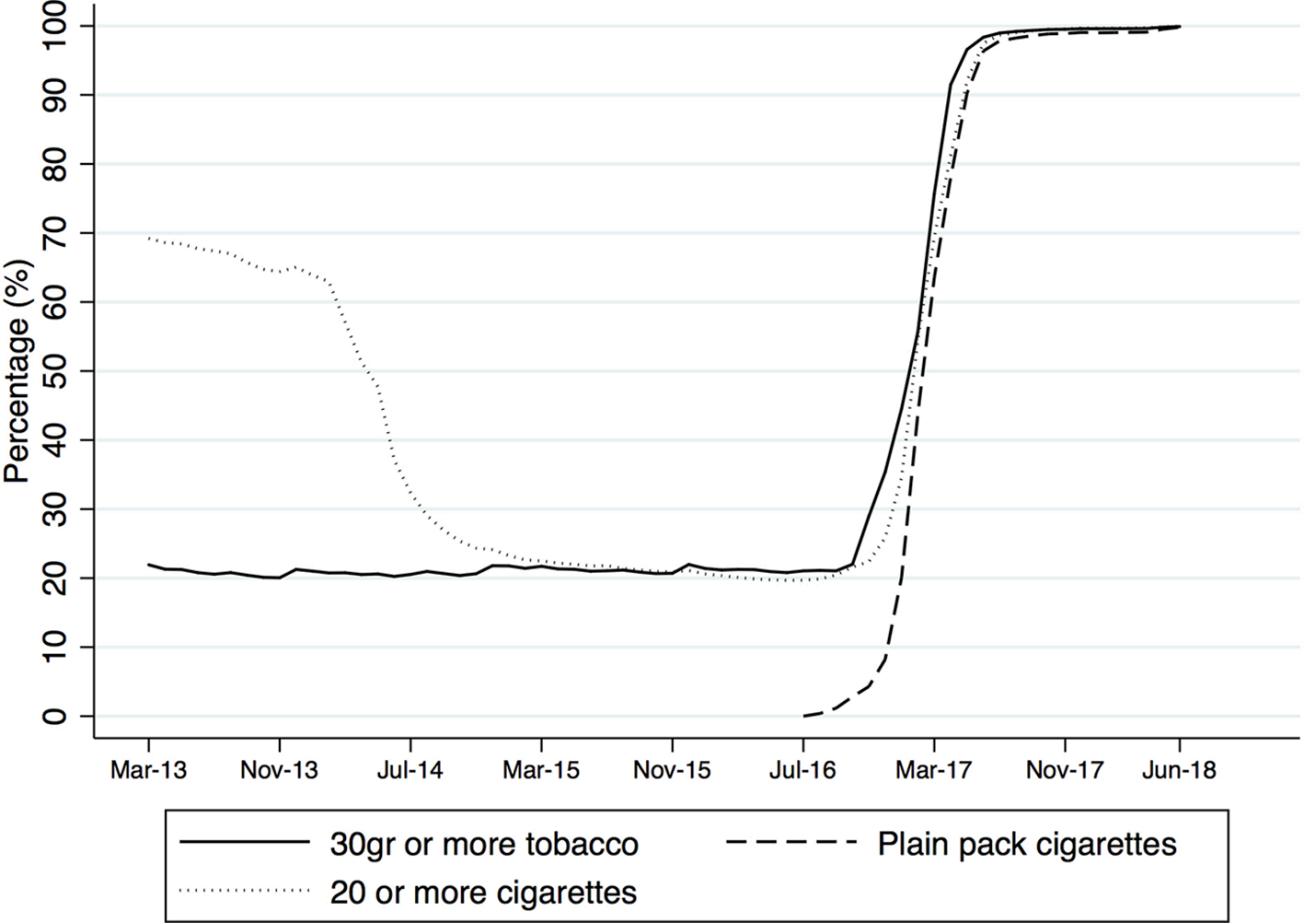
Proportion of roll-your-own tobacco sales in 30 g or more and cigarette sales in plain packs and 20 cigarettes per pack or more, March 2013–June 2018.

The rapid increase in the proportion of sales volume in pack sizes that were legal under the new legislation occurred almost simultaneously for both cigarettes and RYO (figure 3), reaching 98% in June 2018 for both product types. This suggests that the proxy variable for plain packs (ie, 30 g packs) was likely to measure the implementation accurately.

#### Regression analysis

From March 2013 until May 2015, the average inflation-adjusted price increased by 1.54 pence per cigarette and 4.42 pence for 1 g of RYO. Our regression analysis demonstrated that for both RYO and cigarettes, prices rose significantly from the beginning of the sell-off period when observing only legal pack size products. When plain pack sales accounted for more than 75% of the total sales, inflation-adjusted prices in plain packs were higher by 12% for RYO (from 34.9 to 38.8 pence per gram, mean difference 4.26, 95% CI 3.99 to 4.53 pence) and by 7% for cigarettes (from 38.6 to 41.13 pence per cigarette, mean difference 2.53, 95% CI 2.24 to 2.83 pence; table 1).

**Table 1:** Difference in average inflation-adjusted price (in pence) during and after the implementation of the plain packaging legislation measured through the proportion (%) of sales volume in plain packaging* for RYO and cigarettes, May 2015–May 2018†

	RYO (price per gram in pence)		Cigarettes (price per cigarette in pence)	
	All products	Only legal pack size	All products	Only legal pack size
Observations	5972	1713	18 062	8246
Average inflation-adjusted price before May 2016 (SD)	33.6 (7.8)	34.5 (5.7)	38.9 (10.8)	38.6 (8.8)
Regression results				
<50% plain pack sales (p value) (95% CI)	−0.29 (0.407) (−0.97 to 0.39)	1.02 (<0.001) (0.48 to 1.56)	1.03 (0.001) (0.39 to 1.167)	1.09 (<0.001) (0.81 to 1.37)
50%–75% plain pack sales (p value) (95% CI)	−0.32 (0.471) (−1.20 to 0.56)	2.07 (<0.001) (1.52 to 2.62)	1.32 (0.003) (0.44 to 2.19)	1.31 (<0.001) (0.92 to 1.70)
>75% plain pack sales (p value) (95% CI)	2.26 (<0.001) (1.22 to 3.31)	4.26 (<0.001) (3.99 to 4.53)	2.22 (<0.001) (1.64 to 2.80)	2.53 (<0.001) (2.24 to 2.83)
Product size	Yes	No	Yes	No
Brand identifier	No	Yes	No	Yes
Tax period fixed effect	Yes	Yes	Yes	Yes

*Reference category in our regression is ‘before May 2016’, from May 2015 to May 2016.

†Based on proportion plain pack volume of sales for cigarettes and inferred plain pack volume of sales for hand-rolling tobacco based on sales of products in 30 g or more.

RYO, roll-your-own tobacco.

## Discussion

This study explores for the first time the effect of the UK plain packaging and minimum pack size legislation, introduced together over a 1-year period between May 2016 and May 2017, on RYO sales volumes and prices. The study demonstrates that in contrast to cigarettes, RYO sales volumes rose progressively, as did prices, and that across a range of products marketed in different pack sizes, sales prices were very similar. This contrasts with the marked diversity in prices across different pack sizes in the cigarette market before the new legislation was introduced, and a secular decline in cigarette sales. We also show that by the time plain packs accounted for 75% or more of sales, while direct price comparison with unbranded packs was still possible, the transition to plain packs and minimum pack sizes was associated with tax-adjusted increases in prices for both RYO and cigarettes.

The data used for this analysis are taken from *Nielsen Scantrack*, a highly representative source of sales volumes and prices from a range of retailers that has been used extensively in the past to investigate tobacco prices and brand diversity.^8 14–16^ Use of these data to explore the effects of plain packaging on cigarette prices was facilitated by the availability of a variable distinguishing plain from fully branded cigarette packs^8^ but the same facility was not available for RYO. However, the simultaneous legislative imposition of a minimum pack size of 30 g overcomes this problem, since 30 g products were almost non-existent in the UK before the new legislation came into force. We were therefore able to use sale in packs of 30 g or more as a proxy for plain packaging, on the assumption that the small proportion of total sales volumes in packs of more than 30 g was switched to plain packs in approximate tandem with the emergence of 30 g packs. The apparently near simultaneous progress in adopting plain pack size for both cigarettes and RYO in our study (figure 3) supports this assumption. Although Nielsen Scantrack includes data from a sample of supermarkets, small shops and convenience stores, online sales of tobacco products is not captured. However, we have no reason to suspect that our findings would be markedly different if data on online sales were included as we have previously demonstrated that prices from online and offline sources are comparable.^10^

That sales volumes for RYO continued to rise throughout the study period while cigarette sales were falling reflects a long-standing trend whereby price-conscious smokers respond to higher cigarette prices either by quitting tobacco use or by switching to the much less expensive option of smoking hand-rolled cigarettes.^9^ Our findings do suggest however that prices for both RYO and cigarettes rose with the full adoption of plain packaging, and it is not clear whether this was the result of manufacturers passing on the costs of changing to plain packs to the consumer, or whether manufacturers used plain packs as a vehicle for higher prices, perhaps as a means to offset the impacts of reduced consumption of cigarettes in plain packs.^17 18^ However, the fact that 30 g packs for RYO were introduced at lower prices initially (when volume of sales was close to zero) suggests that this could have been an attempt by the industry to allow costumers to adjust to the new upfront cost of buying a larger pack. The introduction of plain packaging in Australia also resulted in higher inflation-adjusted hand-rolling and cigarette prices, although the increase for the former was 1.9 cents per 0.8 g tobacco (2.4 cents per gram),^7^ which is 1.3 pence per gram in UK prices and hence smaller in real terms, despite the approximate 5-year time difference, in magnitude to that in our study.

Our study thus demonstrates that the introduction of plain packs in the UK has resulted in higher tobacco prices, which would be expected to translate into lower consumption.^19^ However the sustained price differentials between manufactured and RYO cigarettes inevitably reduce that effect, particularly since one hand-rolled cigarette typically contains between 0.5 g and 0.75 g of tobacco.^20 21^ RYO thus remains a much less expensive and still increasingly used means of continuing to smoke despite rising costs. Also, the fact that there are still promotional prices in the data set (defined as a price reduced by 5% or more from the second highest price for the same product observed in the preceding 6 weeks) suggests that tobacco control policy could be further strengthened by implementing bans on price discounting. Further studies of market segmentation are necessary to determine whether these price trends apply across all price categories within the RYO market, or whether the practice of overshifting tax onto premium brands that has been well documented for cigarettes in the past^15^ is being applied to RYO products today. Also, detailed analysis of changes in volume of sales in response to plain packaging policy and minimum excise duty implementation by price categories would inform policy makers about the separate and cumulative effects of these policies. Nonetheless, it remains to be seen whether the introduction of plain packaging in other countries results in similar upward trends in prices, and whether there is any appreciable independent long-term effect on smoking prevalence.

**What this paper adds**

- There is little evidence about the effects of plain packaging legislation outside Australia.
- There is no evidence on the effects of plain packaging legislation on roll-your-own (RYO) tobacco market and pricing in the UK.
- Our results show that plain packaging and minimum pack size pack legislation were associated with an increase in tobacco price, and that price of RYO products increased more than the price of cigarettes.
